# Quality, source transparency, information reliability, and domain coverage of short video-based information on feeding guidance for people with dysphagia: a cross-sectional study on 4 major Chinese social media platforms

**DOI:** 10.3389/fdgth.2026.1919212

**Published:** 2026-10-14

**Authors:** Jiaying Chen, Ting Xiong, Ying Li, Yue Wang, Chen Yang, Jinghui Huang, Fanfu Fang

**Affiliations:** 1 Department of Rehabilitation Medicine, The First Affiliated Hospital of Naval Medical University, Shanghai, China; 2 Child Health and Rehabilitation Clinic, Department of Pediatrics, Qilu Hospital of Shandong University, Jinan, China; 3 Department of Rehabilitation Medicine, Shuguang Hospital Affiliated to Shanghai University of Traditional Chinese Medicine, Shanghai, China

**Keywords:** dysphagia, feeding behavior, information quality, short video, social media

## Abstract

**Background:**

Standardized feeding guidance is valuable for people with dysphagia, helping prevent aspiration, improve nutritional status, and enhance quality of life. In recent years, short-video platforms have become a key avenue for the public to access health information, providing people with dysphagia with a convenient channel to learn about feeding management. However, the quality of online health information varies considerably, making it difficult for patients to effectively distinguish reliable information from potential misconceptions, which may negatively impact clinical outcomes.

**Objective:**

This study aims to systematically evaluate feeding guidance content for dysphagia on mainstream Chinese short-video platforms by analyzing its quality, the transparency of its information sources, the reliability of the information, and its coverage of relevant domains.

**Method:**

This study retrieved and included 278 short videos on feeding guidance for dysphagia as research samples from four major Chinese mainstream short video platforms: Rednote, Bilibili, Douyin, and Kwai. Two independent rehabilitation therapists specializing in dysphagia rehabilitation evaluated the domain coverage of video content using 10 criteria derived from relevant clinical guidelines and expert consensus documents on feeding guidance for people with dysphagia. We assessed the transparency of the video's information sources, overall quality, and information reliability using the Journal of the American Medical Association (JAMA) Benchmark Criteria, the Global Quality Score (GQS), and the modified DISCERN (mDISCERN) Score. We also compared video data from different content providers and upload platforms.

**Results:**

Based on the identity of the video uploader, videos were classified into seven categories, comprising four categories of healthcare professionals (physiatrists, rehabilitation therapists, rehabilitation nurses, and healthcare professionals in related clinical departments) and three categories of non-healthcare professionals (non-profit organizations, individual science communicators, and people with dysphagia). The results indicated that the overall quality of feeding guidance videos for people with dysphagia was generally low (GQS median = 2, IQR = 2–3). The impact of uploader identity varied across assessment dimensions: healthcare professionals scored significantly higher than non-healthcare professionals on both the JAMA score (*P* < .001) and the mDISCERN score (*P* < .001), although the inter-group difference in the GQS score was not statistically significant; rehabilitation nurses performed best on the GQS dimension, healthcare professionals in related clinical departments achieved the highest scores on the mDISCERN dimension, and people with dysphagia scored the lowest on the JAMA dimension. Differences among platforms also exhibit dimensionality dependence: the GQS scores showed minimal variation, with only Bilibili and Kwai exhibiting a statistically significant difference (adjusted *P* < .05, *r* = .376); the JAMA scores demonstrated moderate variation, with Rednote, Douyin, and Bilibili all significantly outperforming Kwai (adjusted *P* < .0001, *r* = .387; adjusted *P* < .0001, *r* = .551; adjusted *P* < .05, *r* = .288); the mDISCERN scores showed the most pronounced differences (*ε*^2^ = .503), with Douyin significantly surpassing Rednote, Bilibili, and Kwai (adjusted *P* < .0001, *r* = .771; adjusted *P* < .0001, *r* = .757; adjusted *P* < .0001, *r* = .595). In terms of domain coverage, food adjustment (77%) and swallowing posture adjustment (52%) had relatively high coverage rates. In contrast, high-risk domains such as medication management (4%), nutritional management (21%), and oral care (17%) exhibited severe supply gaps.

**Conclusion:**

Among current mainstream short-video platforms in China, the overall quality of educational content on feeding guidance for people with dysphagia still requires improvement. The reliability of this information depends on both content creators' professional backgrounds and platform ecosystems. The existing short videos currently lack sufficient support for safe decision-making and standardized self-management among people with dysphagia. There is an urgent need to implement coordinated interventions that incorporate professional expertise, platform algorithm governance, and multidimensional quality assessments to support the standardized development of a health science communication environment.

## 1 Introduction

Dysphagia is the inability to safely and effectively transport food from the mouth to the stomach because of structural and/or functional impairment of organs such as the jaw, lips, tongue, soft palate, pharynx, and esophagus (1). Dysphagia can be triggered by various diseases, encompassing central nervous system disorders, peripheral nervous system disorders, muscular diseases, organic lesions of the oropharynx, digestive system diseases, respiratory system diseases, etc (2). Dysphagia can severely impact patients' nutritional intake, quality of life, and prognosis, while significantly increasing the risk of developing conditions such as aspiration pneumonia (3).

Currently, the treatment and rehabilitation management of dysphagia exhibit significant complexity, and single clinical intervention methods often fail to meet the long-term management needs of patients. In this context, daily feeding management is regarded as an important auxiliary support measure (4). Standardized feeding guidance, including adjustments to food texture, intervention in eating posture, and compensatory strategies, is a core component of dysphagia management. It is crucial for ensuring patient feeding safety and improving nutritional status (5). Research indicates that people with dysphagia and their caregivers have an urgent need for standardized feeding guidance. However, because professional rehabilitation resources are scarce and poorly accessible, patients often lack guidance (6).

The widespread adoption of the Internet has brought unprecedented convenience in accessing health information. Even before healthcare professionals paid substantial attention to social media as a venue for health popularization, patient groups had already become active on various online platforms to seek medical-related support and information. In recent years, people with dysphagia and their caregivers have increasingly preferred to obtain feeding management advice from social media rather than traditional medical channels (7). Among these platforms, short-video platforms like Douyin and Rednote have rapidly grown in health information dissemination because of their intuitive presentation, ease of sharing, and high interactivity (8). Compared with text or static images, videos can more vividly demonstrate feeding techniques, helping learners imitate and retain information, thereby supporting patients and caregivers in self-management.

However, the open nature of the online information environment leads to widely varying content quality. Numerous videos concerning feeding guidance for dysphagia are posted by individual users without professional medical training, leading to information that may be inaccurate, incomplete, or even misleading. Inappropriate feeding advice not only fails to help but may also increase serious risks such as aspiration, potentially harming patient health. For most users without professional backgrounds, assessing the reliability and quality of these dietary recommendations can be quite challenging. Although studies have shown (9) that a significant proportion of patients regard online information as their primary source of credibility, the ambiguity of information producers’ professional identities casts doubt on the content's scientific validity and reliability.

China has a vast number of short-video users, enabling people with dysphagia and their caregivers to conveniently access educational content on eating through platforms such as Douyin, Rednote, Bilibili, and Kwai. Consequently, existing domestic research has primarily focused on the effects of digital media as carriers for rehabilitation interventions, particularly using short videos to deliver oral and facial muscle training, laryngeal exercises, sensory stimulation, and safe swallowing technique instruction (10–13). Scholars in this field have yet to establish a comprehensive framework for dietary guidance, nor have they conducted systematic quality assessments of the existing educational content on dysphagia available on public social platforms.

Therefore, this study used a cross-sectional design and systematically incorporated feeding guidance videos for people with dysphagia from four major platforms—Rednote, Douyin, Bilibili, and Kwai. Using the GQS, JAMA, and mDISCERN tools, we evaluated content quality, source transparency, and information reliability, respectively, and then developed a domain-coverage framework for dysphagia feeding guidance. Compared to previous broad-based swallowing intervention studies that focused primarily on oral muscle training and demonstrations of safe swallowing techniques, this study no longer merely aimed to verify whether educational videos can be used in rehabilitation teaching. Instead, it audited the coverage and quality of feeding guidance content across real-world platform ecosystems, aiming to identify gaps between science popularization content on short-video platforms and clinical safety standards. This approach provides differentiated evidence-based support for clinicians in recommending authoritative, certified educational resources, for social media platforms in implementing tiered content moderation systems, and for algorithmic quality governance initiatives.

## 2 Methods

### 2.1 Retrieval strategy and data extraction

We sourced all collected videos from four major Chinese short-video platforms: Rednote, Douyin, Bilibili, and Kwai. Search keywords included “dysphagia” or “swallowing” combined with “feeding,” “diet,” or “nutrition.” The entire search process was completed between September 21 and September 23, 2025, using native apps on domestically produced Android smartphones. The device's geographic location was set to Shanghai. However, before the search, we disabled the phone's location services and blocked all apps from accessing location data. To minimize the impact of personalized recommendations, we created new accounts on each of the four platforms and, after registration, set these accounts to zero views, zero searches, and zero saved-item history. We conducted searches sequentially on each platform using the following six keyword combinations: ① dysphagia, feeding; ② dysphagia, diet; ③ dysphagia, nutrition; ④ swallowing, feeding; ⑤ swallowing, diet; ⑥ swallowing, nutrition. From each set of results, we selected the top 200 cross-sectional samples from each platform's search and recommendation results, yielding 800 initial samples across the four platforms. The results were recorded in descending order according to the default display sequence of each app (combined/recommendation sorting), without manual switching to “latest” or reordering by popularity; no searches were repeated.

We included only Chinese videos primarily focused on feeding guidance for dysphagia. Exclusion criteria included duplicate videos, irrelevant content, non-Chinese videos, videos with blurry images or poor audio quality, videos for commercial use, and videos where the creator's identity could not be verified. Additionally, cases of dysphagia resulting from behavioral abnormalities due to cognitive or psychiatric disorders were excluded (Figure 1). We extracted basic information from the included videos, including video title, identity, video duration, number of likes, number of comments, number of favorites, number of shares, and the number of days since the initial upload. All extracted data is recorded in Excel.

**Figure 1:**
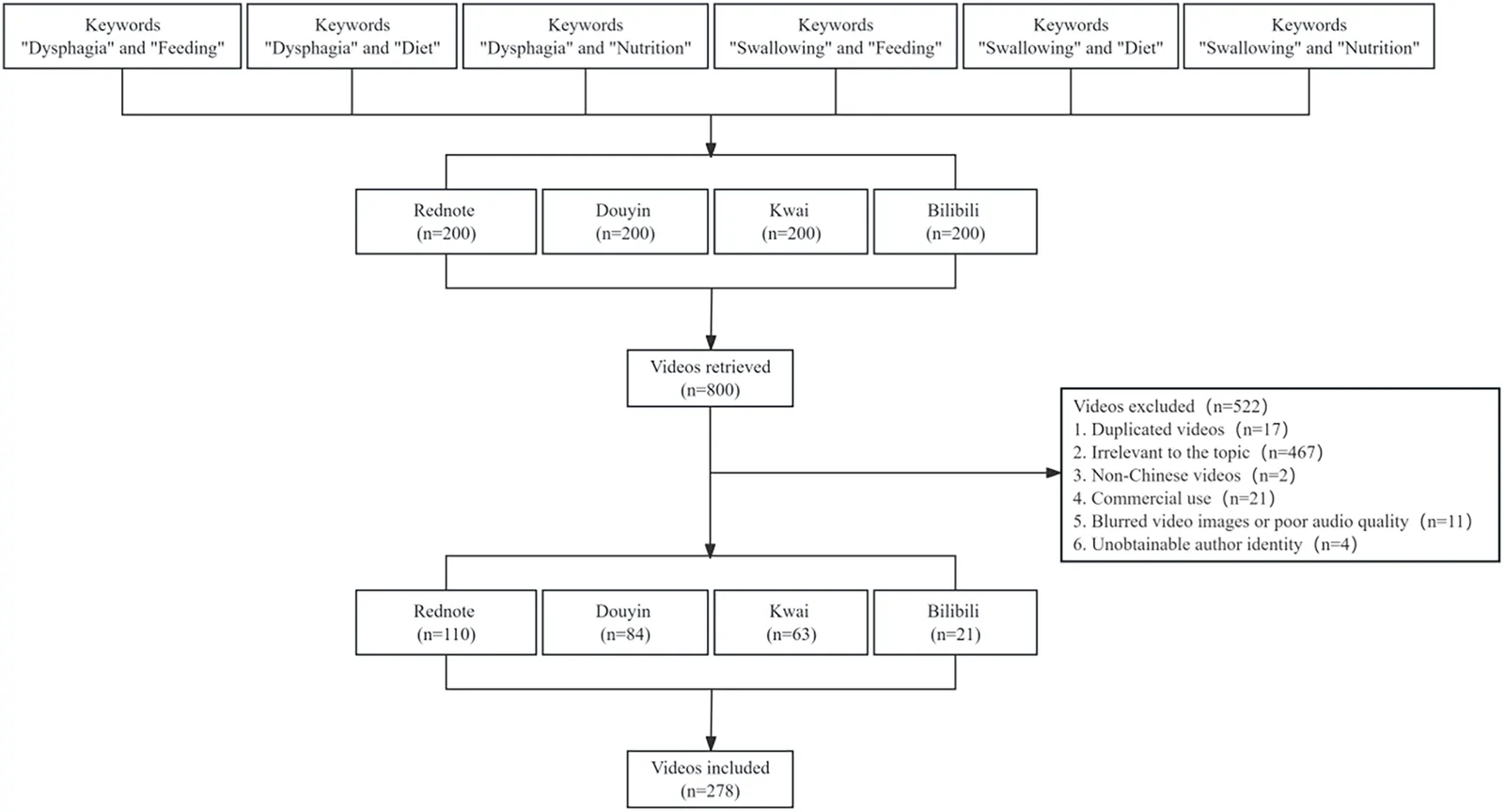
Search strategy and video screening procedure.

### 2.2 Evaluation method

The coverage of feeding guidance for dysphagia is based on the latest recommendations from the American Speech-Language-Hearing Association (ASHA) and the best available evidence from China and internationally regarding feeding guidance for dysphagia (14–20) (including clinical guidelines and expert consensus statements), and is summarized in the following 10 domains: adjustment of feeding environment, adjustment of feeding methods, adjustment of feeding utensils, adjustment of food, adjustment of swallowing posture, feeding movement techniques, feeding risk control, oral care, nutritional management, and medication management. Specific guidance recommendations are detailed in (Table 1). In this study, the domain coverage score is defined as the domain mention rate across the aforementioned domains, with each domain mentioned in the video contributing 1 point. This metric solely reflects the breadth of content coverage and is not used as a criterion for assessing content quality thresholds.

**Table 1:** Evaluation of detailed contents of feeding guidance for dysphagia.

Aspects of feeding guidance	Recommendations
Adjustment of Feeding Environment	Reduce distractions, lower noise levels, enhance lighting, promote social interaction, and increase favorable feeding cues. (e.g.verbal cues, written cues and signs, physical cues, visual cues, etc.)
Adjustment of Feeding Methods	Oral feeding, enteral nutrition (continuous tube feeding and its nursing care, intermittent orogastric/esophageal tube feeding and its nursing care, fistulization care).
Adjustment of Feeding Utensils	Use modified utensils during feeding, including spoons, bowls, chopsticks, forks, cups, straws, syringes, etc.
Adjustment of Food	Adjustment of food consistency. (e.g.thickening liquids with thickeners; using food with appropriate texture levels based on the results of swallowing and feeding assessment)
	Adjustment of food bolus size. (e.g. Using a safe food bolus size based on the results of swallowing and feeding assessment.)
Adjustment of Swallowing Posture	Adjustment of trunk posture during swallowing. (e.g.), semi-recumbent trunk position, upright trunk position, lateral trunk tilt position, etc.)
	Adjustment of head and neck posture during swallowing. (e.g.), head-neck extension, head-neck flexion, head-neck rotation, etc.).
Feeding Movement Techniques	Chin-tuck swallowing, chin-up swallowing, chin-up → chin-tuck swallowing, head-turn swallowing, lateral swallowing, nose-pinching swallowing, multiple swallowing, effortful swallowing, alternating swallowing, etc.
Feeding Risk Control	Monitor and accompany the patient during feeding; observe changes in the patient's consciousness, respiration, blood oxygen saturation, etc., during feeding. Management of emergencies such as choking, aspiration, and asphyxia. (e.g., Heimlich maneuver, cough, and sputum excretion techniques).
Oral Care	Maintain the oral mucosa, teeth, gums, tongue, buccal grooves, and pharynx in a comfortable, clean, moist, and infection-free state through various methods, including gargling, negative-pressure irrigation, toothbrushing, cold-hot oral brushing, and traditional specialized oral care (e.g.,oral care for patients with tracheotomy), etc.
Nutritional Management	Enteral nutrition preparations, oral nutritional supplements, or formula foods for special medical purposes.
Medication Management	Under the guidance of physicians or pharmacists, crush medications, dissolve them in water, or mix them with safely swallowable food for administration.

In this study, JAMA, GQS, and modified DISCERN (mDISCERN) were used to assess the transparency, overall quality, and reliability of feeding guidance information provided by short videos for people with dysphagia, respectively. The JAMA (21) provides an objective benchmark for assessing the credibility and transparency of information, serving as the foundation for the traceability and verifiability of academic content. Grounded in core academic ethical principles, they evaluate video sources, author qualifications, timeliness, and conflict of interest disclosures [Appendix 1]. The scoring range for the JAMA Standards is defined as the total number of accurately recommended dimensions across the above criteria, with a maximum possible score of 4 points. The mDISCERN scale (22) aims to conduct an in-depth, structured analysis of information content quality. Aligned with evidence-based medicine principles, it thoroughly evaluates the clarity, relevance, traceability, robustness, and impartiality of video content [Appendix 2]. The mDISCERN scale scores range from 0 to 5 points, representing the total number of accurate recommendations across these dimensions. As a comprehensive assessment tool (23), GQS evaluates users' (e.g., people with dysphagia in this study) subjective, intuitive overall perception of information quality, including clarity, logical organization, comprehensibility, and overall credibility impression [Appendix 3]. The GQS score provides a crucial metric for measuring the effectiveness of information dissemination and potential users' engagement, thereby addressing the shortcomings of the aforementioned objective and structured tools. The GQS scoring range is defined as the total number of accurate recommendations across all dimensions, with a maximum possible score of 5 points.

### 2.3 Evaluation process

Two rehabilitation therapists (CJY and XT), who hold national qualifications and have extensive clinical experience, conducted the assessments; both practitioners work in the department of rehabilitation medicine at a tertiary teaching hospital. All videos were browsed without downloading, forwarding, liking, or commenting.

Prior to scoring the videos, the two raters reviewed the feeding guidance guidelines for people with dysphagia, as well as the official scoring criteria from JAMA, GQS, and mDISCERN; they then discussed how to apply these tools to evaluate the video content and made necessary adjustments. Both raters (CJY and XT) assessed each video; when they disagreed, a third rater (LY) was consulted to reach consensus. For scores where no consensus could be reached, the final decision was made by the senior author (FFF or HJH). We quantified inter-rater reliability using Cohen's *κ* coefficient. The inter-rater reliability for each assessment item exceeded 0.8, indicating good inter-rater reliability [Appendix 4].

All statistical analyses were performed using GraphPad Prism 10.1 and SPSS 23.0, with a significance level of *α* = 0.05; all tests were two-sided. We conducted overall comparisons among three or more independent samples using the Kruskal–Wallis H test, with the overall effect size reported as *ε*^2^, calculated as *ε*^2^ = H/(N_total_ − 1). When the Kruskal–Wallis overall test indicated a statistically significant difference, we used the Dunn test for *post-hoc* pairwise comparisons, applying a Bonferroni correction to control the familywise error rate. For all other pairwise comparisons among independent groups, we used the Mann–Whitney *U* test and applied the Benjamini–Hochberg false discovery rate (FDR) correction for multiple comparisons. The effect size r for all pairwise comparisons was calculated using the formula $r=|Z|/\sqrt{N_{\mathrm{total}}}$, where Z denotes the Z-statistic obtained from the Dunn test or the Mann–Whitney *U* test, and N_total_ represents the total sample size; the 95% confidence interval for the effect size r was then estimated using the Fisher z-transformation.

## 3 Results

### 3.1 Video characteristics

After applying the inclusion and exclusion criteria, this study included 278 video samples for content analysis (Figure 1). Researchers conducted a search using six sets of preset keywords on four Chinese short-video platforms: Rednote, Douyin, Bilibili, and Kwai, initially obtaining 800 relevant videos. Next, we screened the initial sample against predetermined inclusion and exclusion criteria: we excluded 17 duplicate videos, 467 videos unrelated to the theme, 2 non-Chinese videos, 11 videos with blurry images or poor audio quality, 21 videos for commercial use, and 4 videos with unidentifiable authors. Ultimately, 278 videos meeting all criteria were included for subsequent data extraction and in-depth analysis. This screening process excluded redundant, irrelevant, low-quality, and unidentified videos, ensuring the final sample set's rigor in thematic relevance, content clarity, and unidentified videos. This approach provided a basis for subsequent in-depth analysis of the health communication characteristics of feeding guidance for people with dysphagia on Chinese short-video platforms.

As shown in Figure 2, from 2019 to 2025, all four short-video platforms saw an increase in videos uploaded on feeding guidance for dysphagia. Among them, Rednote and Douyin grew more significantly than Bilibili and Kwai. Rednote ranked first among the four platforms in terms of video uploads.

**Figure 2:**
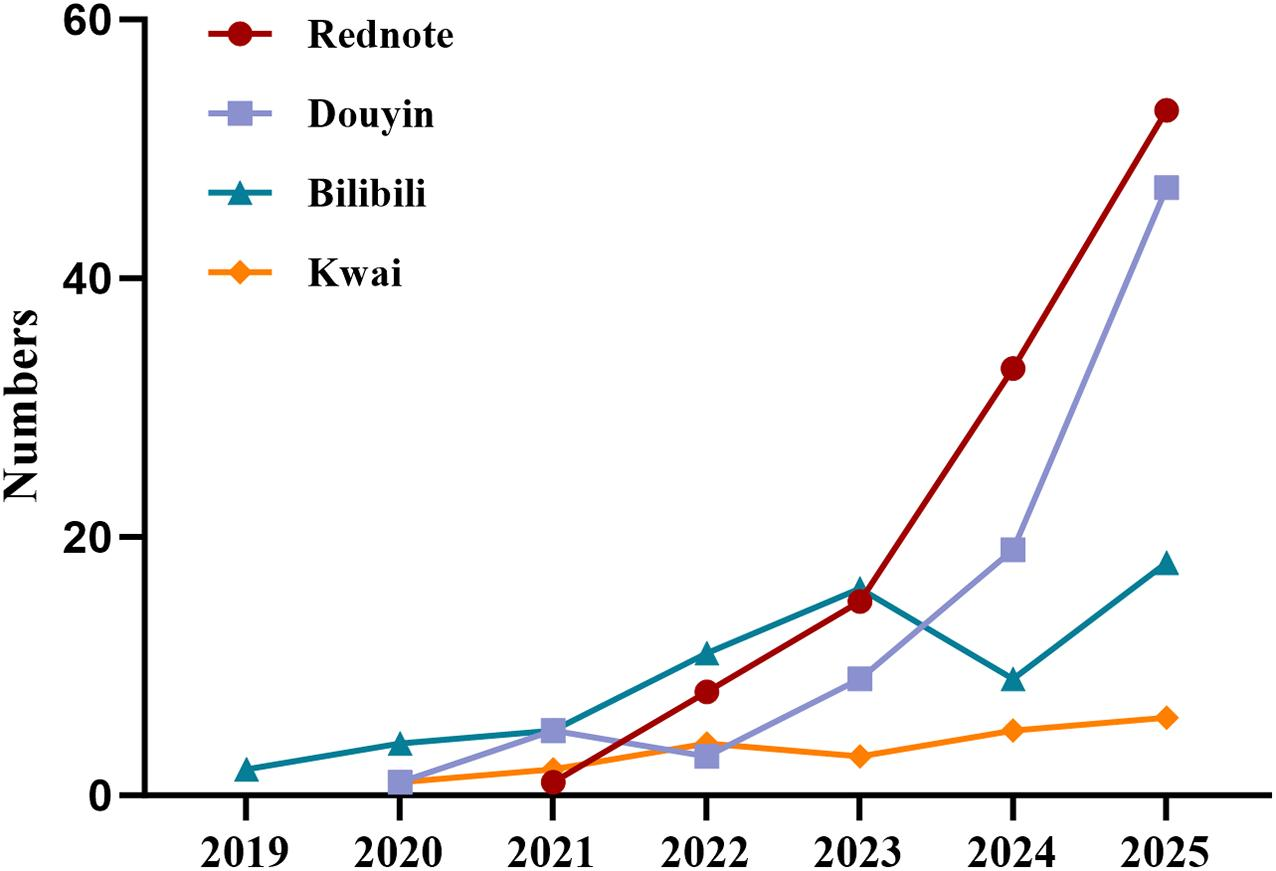
Trend of video upload quantity over the years.

We categorized the 278 screened videos into two groups based on the uploader's identity: healthcare professionals and non-healthcare professionals. Further classification reveals that healthcare professionals can be categorized into physiatrists (doctors who specialize in physical medicine and rehabilitation), rehabilitation therapists (professionals who provide therapeutic interventions to restore function), rehabilitation nurses (nurses who specialize in the care of patients undergoing rehabilitation), and healthcare professionals in related clinical departments (excluding those from the rehabilitation department); non-healthcare professionals can be categorized into non-profit organizations (organizations operating for charitable support and advocacy), individual science communicators (independent creators of publicly accessible health education content), and people with dysphagia.

As shown in Table 2, 178 of the 278 videos (64%) were uploaded by healthcare professionals, while 100 (36%) were uploaded by non-healthcare professionals. Among healthcare professionals, the highest number of videos were uploaded by those in related clinical departments (*n* = 92, 33%). Among rehabilitation medicine practitioners, the order of uploaded videos from most to least is as follows: rehabilitation therapists (*n* = 39, 14%), physicians (*n* = 32, 12%), and rehabilitation nurses (*n* = 15, 5%). Among non-healthcare professionals, the highest number of videos were uploaded by individual science communicators (*n* = 61, 22%), followed by non-profit organizations (*n* = 29, 10%) and people with dysphagia (*n* = 10, 4%).

**Table 2:** Characteristics of the videos across sources.

Identity of the video uploader	Duration median (IQR)	Like count median (IQR)	Comment count median (IQR)	Bookmark count median (IQR)	Share count median (IQR)	Videos *n* (%)
Overall	102 (57–199)	35 (8–103)	1 (0–6)	21 (5–91)	16 (2–57)	278 (100)
Healthcare professionals	99 (52–190)	36 (11–98)	2 (0–6)	23 (5–94)	17 (4–55)	178 (64)
Physiatrists	95 (68–179)	45 (18–115)	3 (1–8)	43 (17–140)	20 (8–63)	32 (12)
Rehabilitation therapists	67 (41–113)	65 (25–185)	3 (0–8)	46 (27–124)	34 (12–59)	39 (14)
Rehabilitation nurses	145 (104–239)	18 (6–126)	0 (0–0)	20 (5–113)	13 (3–143)	15 (5)
Related Clinical Departments	104 (50–190)	27 (9–75)	2 (0–4)	9 (3–38)	11 (1–38)	92 (33)
Non-healthcare professionals	120 (63–227)	31 (6–117)	1 (1–6)	17 (4–87)	12 (1–59)	100 (36)
Non-Profit Organizations	103 (61–194)	52 (10–126)	1 (0–5)	31 (6–99)	32 (3–101)	29 (10)
Individual Science Communicators	125 (73–257)	11 (2–58)	0 (0–2)	15 (4–87)	7 (1–62)	61 (22)
People with Dysphagia	46 (40–179)	112(80–272)	21(13–44)	14(7–63)	8(3–22)	10(4)

For all included videos, the median duration was 102 s (IQR: 57–199 s), the median number of likes was 35 (IQR: 8–103), the median number of comments was 1 (IQR: 0–6), the median number of saves was 21 (IQR: 5–91), and the median number of shares was 16 (IQR: 2–57). Further analysis revealed that videos uploaded by healthcare professionals were shorter in duration (median 99 s, IQR: 52–190 s), but they received more likes (median 36, IQR: 11–98), comments (median 2, IQR: 0–6), favorites (median 23, IQR: 5–94), and shares (median 17, IQR: 4–55) than those uploaded by non-medical professionals.

Furthermore, videos were categorized into four types by upload platforms. Of the 278 videos, 110 originated from Rednote (40%), making it the platform with the most videos, while Kwai had only 21 videos (7%), making it the platform with the fewest. The number of likes was highest on Rednote (median: 43; IQR: 6–124); the number of comments was highest on Douyin (median: 3; IQR: 0–7); the number of shares was highest on Bilibili (median: 32; IQR: 8–95); the number of bookmarks was highest on Bilibili (median: 48; IQR: 16–217); and the video duration was longest on Bilibili (median: 283; IQR: 167–791 s) (Table 3).

**Table 3:** Characteristics of the videos across video platforms.

Video platform	Duration median (IQR)	Like count median (IQR)	Comment count median (IQR)	Bookmark count median (IQR)	Share count median (IQR)	Videos *n* (%)
Rednote	85 (54–129)	43 (6–124)	1 (0–6)	34 (6–106)	27 (3–59)	110 (40)
Douyin	66 (46–114)	42 (16–123)	3 (0–7)	10 (4–33)	9 (2–34)	84 (30)
Bilibili	283 (167–791)	17 (4–63)	1 (0–3)	48 (16–217)	32 (8–95)	63 (23)
Kwai	122 (58–208)	36 (19–52)	1 (0–7)	5 (0–17)	0(0–12)	21(7)

Regarding the distribution of participants across the four platforms, only Douyin and Rednote include all seven categories of content uploaders; Kwai includes only videos uploaded by healthcare professionals in related clinical departments, non-profit organizations, and individual science communicators. Bilibili, however, lacks videos uploaded by rehabilitation nurses and people with dysphagia. On both Douyin and Kwai, healthcare professionals in related clinical departments account for the most uploads. In contrast, on Rednote, rehabilitation therapists have the most uploads, and on Bilibili, individual science communicators contribute the most videos (Figure 3).

**Figure 3:**
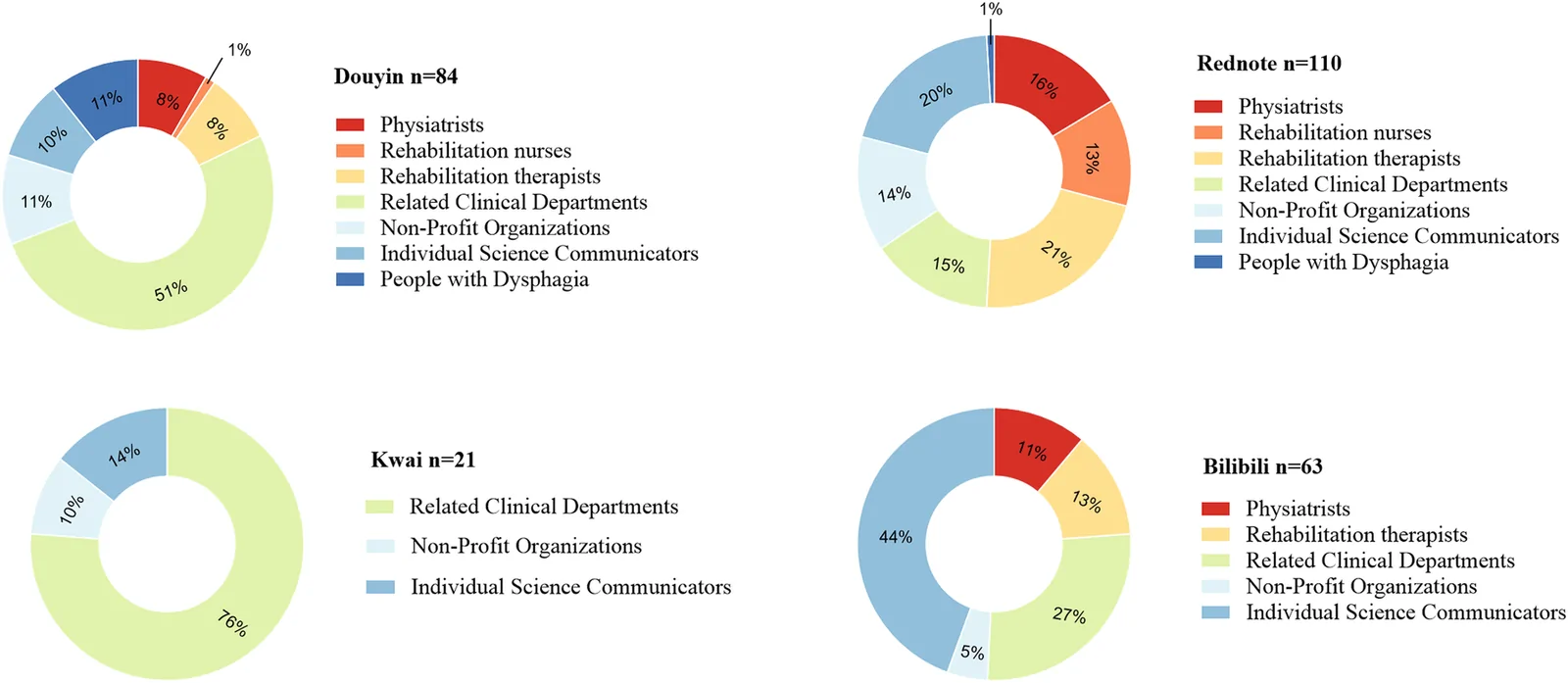
Donut chart of different personnel proportions on four platforms.

### 3.2 Video content

The domain coverage of 278 videos reveals significant, systematic gaps in content within current dysphagia feeding guidance videos. The vast majority of videos focus on isolated knowledge points, with a severe lack of systematic feeding guidance for people with dysphagia. Overall, more than 70% of the videos did not mention the five areas of feeding environment adjustment, feeding risk control, oral care, nutritional management, and medication management. Food texture modification was the most frequently mentioned area (77% coverage rate), while approximately half of the videos addressed swallowing posture adjustments (52% coverage rate) (Table 4).

**Table 4:** Domain-specific evaluation scores for dysphagia feeding guidance [1 point, *n* (%)].

Identity of the video uploader	D1	D2	D3	D4	D5	D6	D7	D8	D9	D10
Overall	25	38	31	77	52	34	26	17	21	4
Healthcare professionals	26	37	31	78	50	42	26	17	17	3
Physiatrists	25	34	16	75	31	37	25	16	9	0
Rehabilitation therapists	10	23	18	59	59	56	26	10	3	3
Rehabilitation nurses	67	53	80	100	80	73	67	53	13	7
Related Clinical Departments	26	40	34	84	48	33	21	14	26	3
Non-healthcare professionals	23	40	32	76	55	19	24	17	27	7
Non-Profit Organizations	28	34	34	82	55	24	41	10	21	7
Individual Science Communicators	23	36	30	70	56	18	20	23	25	2
People with Dysphagia	10	80	40	80	50	10	0	0	60	0

D1, Adjustment of Feeding Environment; D2, Adjustment of Feeding Methods; D3, Adjustment of Feeding Utensils; D4, Adjustment of Food; D5, Adjustment of Swallowing Posture; D6, Feeding Movement Techniques; D7, Feeding Risk Control; D8, Oral Care; D9, Nutritional Management; D10, Medication Management.

Across 10 domains, healthcare professionals more often mentioned “adjustment of feeding environment,” “adjustment of food,” “feeding movement techniques,” and “feeding risk control.” Non-healthcare professionals, conversely, more often referenced “adjustment of feeding methods,” “adjustment of feeding utensils,” “adjustment of swallowing posture,” and “nutritional management.” “Oral care,” and “medication management” were mentioned at similar rates by both groups, but were generally underrepresented (Figures 4A–C). Based on the identity of the uploaders, the overall rating of video content produced by healthcare professionals is slightly higher than that of non-healthcare professionals. However, no significant difference is observed (Figure 4D).

**Figure 4:**
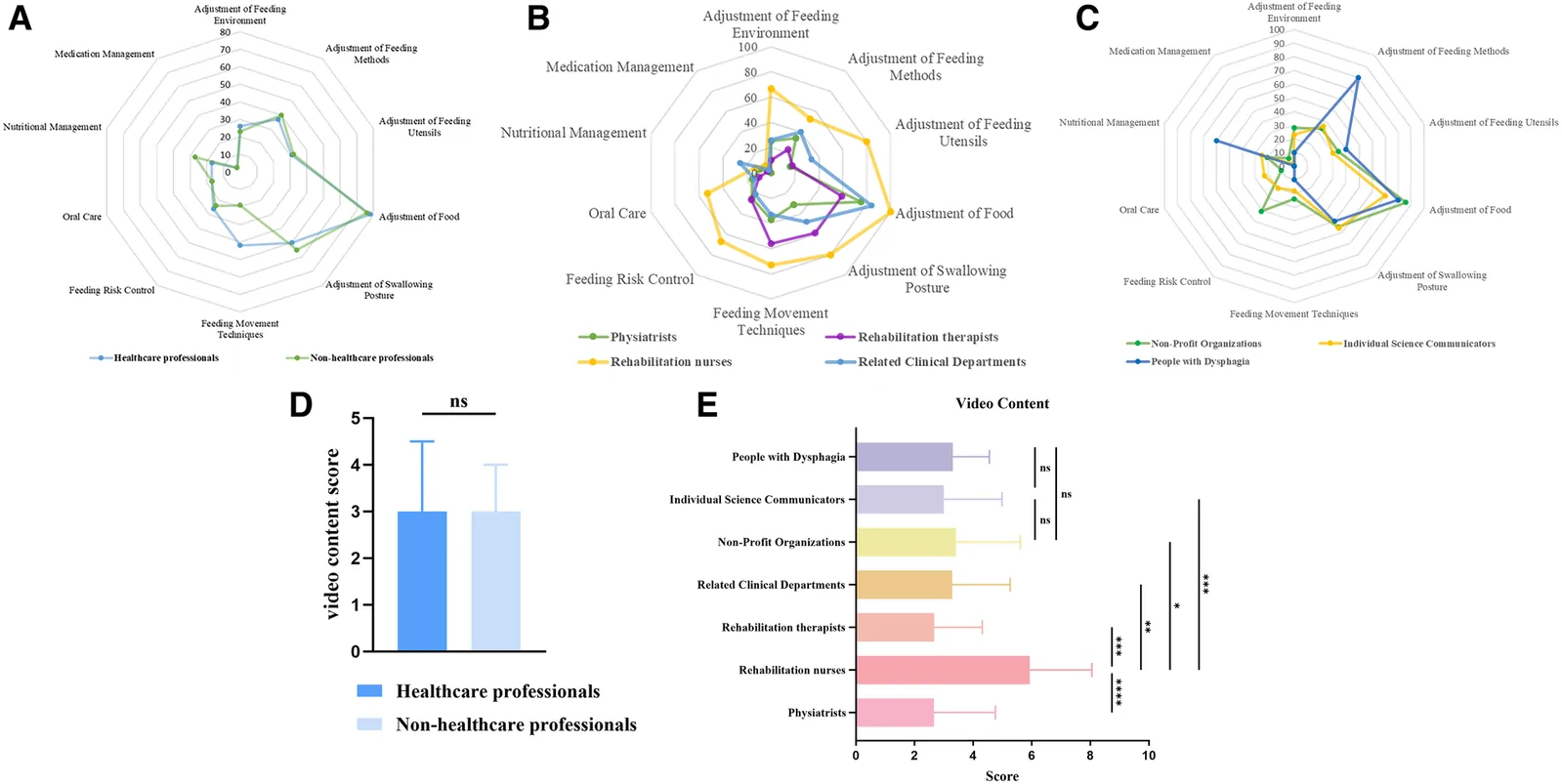
Comparison of content comprehensiveness among different categories of uploaders. **(A–C)** Radar chart shows the percentage of feeding guidance recommendations for dysphagia in videos from different categories of uploaders. **(D,E)** Bar chart shows the total score of video content from different categories of uploaders. *adjusted P < .05; **adjusted P < .01; ***adjusted P < .001; ****adjusted P < .0001; ns: no significant.

Specifically, statistically significant differences were observed across the seven categories of identity (*H* = 25.92, df = 6, *P* = .0002, *ε*^2^ = .094); the domain coverage rate of rehabilitation nurses was significantly higher than that of physiatrists [*adjusted P* < .0001, *r* = .677, 95% CI (0.48, 0.81)], rehabilitation therapists [*adjusted P* *=* .0002, *r* = .608, 95% CI (0.41, 0.75)], healthcare professionals in related clinical departments [*adjusted P* = .0028, *r* = .369, 95% CI (0.19, 0.52)], non-profit organizations [*adjusted P* = .0201, *r* = .498, 95% CI (0.24, 0.69)], and individual science communicators [*adjusted P* *=* .0005, *r* = .483, 95% CI (0.29, 0.64)] (Figure 4E). Rehabilitation nurses achieved the highest score (100%) in the “adjustment of food” domain and the lowest score (7%) in the “medication management” domain. Physiatrists achieved the highest score rate (75%) in the “adjustment of food” domain and the lowest score rate (0%) in the “medication management” domain. Therapists achieved the highest score rate in the “adjustment of food” domain and the “adjustment of swallowing posture” domain (59%), followed by the “feeding movement techniques” domain (56%), while the lowest score rate was observed in the “nutritional management” domain and the “medication management” domain (3%). Healthcare professionals in related clinical departments achieved the highest score rate in the “adjustment of food” domain (84%) and the lowest score rate in the “medication management” domain (3%) (Table 4).

Furthermore, analysis of the overall domain coverage scores across different platforms revealed statistically significant differences (*H* = 9.557, df = 3, *P* = 0.0227, *ε*^2^ = 0.035). Douyin had the lowest domain coverage score (Table 5), significantly lower than Rednote [*adjusted P* = 0.0382, *r* = 0.196, 95% CI (0.06, 0.33)] (Figure 5A). Further analysis indicated that the scores of healthcare and non-healthcare professionals varied across platforms: on Rednote and Bilibili, healthcare professionals scored slightly lower than non-healthcare professionals; on Douyin and Kwai, healthcare professionals scored slightly higher than non-healthcare professionals, though no statistically significant differences were observed between the groups (Table 5; Figure 5B).

**Table 5:** Overall content comprehensiveness scores for dysphagia feeding guidance.

Video platform	Scores		
	Overall median (IQR)	Healthcare professionals median (IQR)	Non-healthcare professionals median (IQR)
Rednote	4 (2–5)	4 (2–5)	4 (2–5)
Douyin	2 (1–4)	3 (2–4)	2 (1–4)
Bilibili	2 (1–5)	2 (1–6)	3 (1–5)
Kwai	2 (2–6)	4 (2–6)	2(1–5)

**Figure 5:**
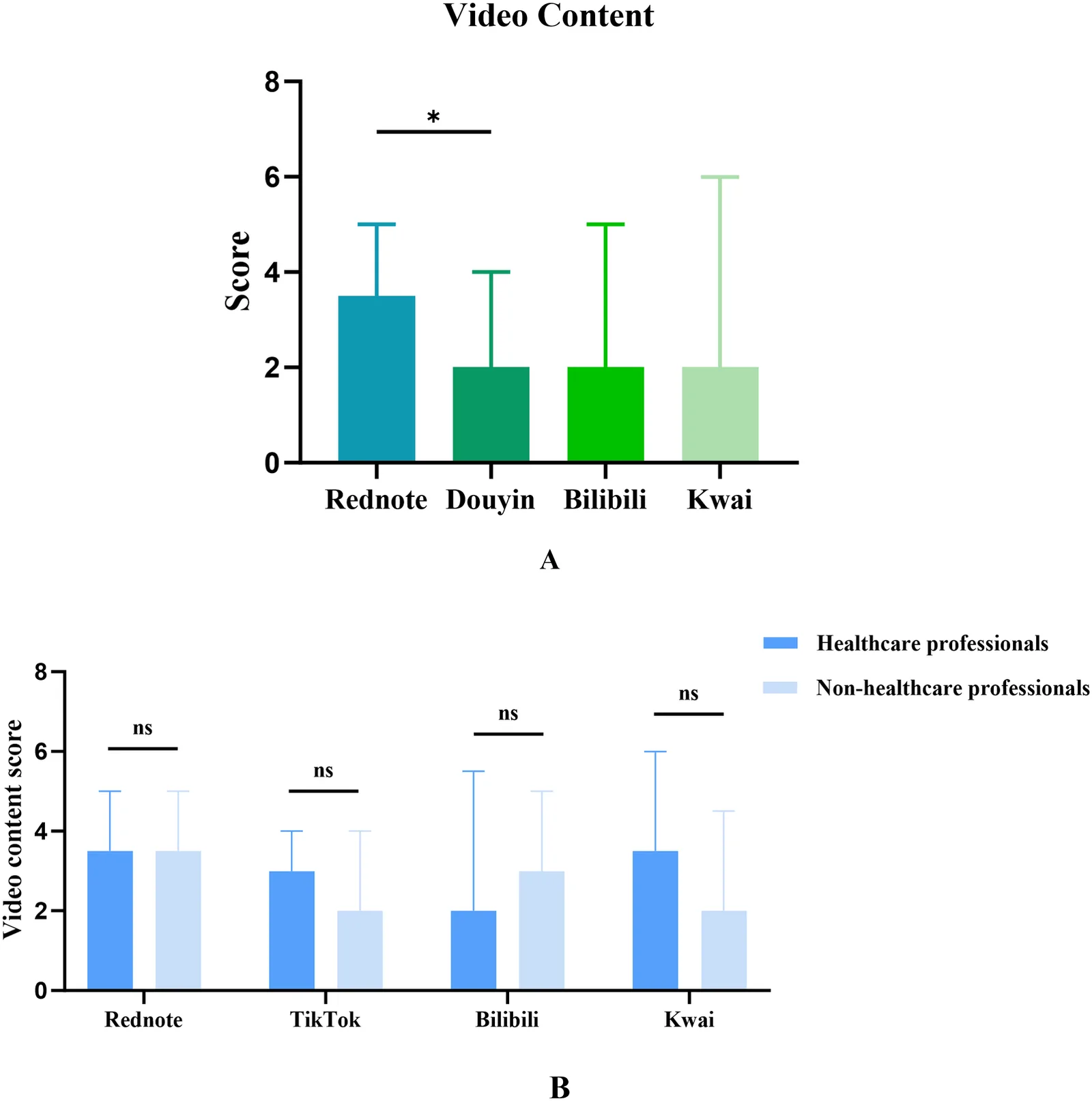
Compare the overall score of video content comprehensiveness across different platforms. **(A,B)** Bar chart compares the total scores of video content for healthcare professionals and non-healthcare professionals from different platforms. *adjusted P < .05; ns: no significant.

### 3.3 Information quality, transparency, and reliability

We used GQS, JAMA, and mDISCERN to assess the overall quality, transparency, and reliability of the source information for each video. As shown in Table 6, the median GQS score for all videos was 2 (IQR: 2–3), the median JAMA score was 3 (IQR: 2–3), and the median mDISCERN score was 3 (IQR: 3–4).

**Table 6:** GQS, JAMA, and mDISCERN ratings of feeding guidance videos for swallowing difficulties based on the uploader's identity.

Identity of the video uploader	GQS scores median (IQR)	JAMA scores median (IQR)	mDISCERN scores median (IQR)
Overall	2 (2–3)	3 (2–3)	3 (3–4)
Healthcare professionals	2 (2–3)	3 (2–3)	3 (3–5)
Physiatrists	2 (2–3)	3 (3–3)	3 (3–4)
Rehabilitation therapists	2 (2–3)	3 (2–3)	3 (3–3)
Rehabilitation nurses	3 (3–4)	3 (3–3)	3 (3–4)
Related Clinical Departments	2 (2–3)	3 (2–3)	4 (3–5)
Non–healthcare professionals	2 (2–3)	2 (1–3)	2 (2–3)
Non-Profit Organizations	2 (2–3)	2 (2–3)	3 (2–4)
Individual Science Communicators	2 (2–3)	2 (1–3)	2 (2–3)
People with Dysphagia	2(2–3)	1(1–2)	3(1–4)

Based on uploader identity, GQS scores were nearly equal for healthcare and non-healthcare professionals, whereas JAMA and mDISCERN scores were significantly higher for healthcare professionals (*P* < .001; *P* < .001; Figure 6A). Further comparisons of the GQS, JAMA, and mDISCERN scores across the seven uploader identities revealed statistically significant differences in overall scores (*H* = 18.15, df = 6, *P* = .0059, *ε*^2^ = .066; *H* = 56.32, df = 6, *P* < .0001, *ε*^2^ = .203; *H* = 56.55, df = 6, *P* < .0001, *ε*^2^ = .204).

**Figure 6:**
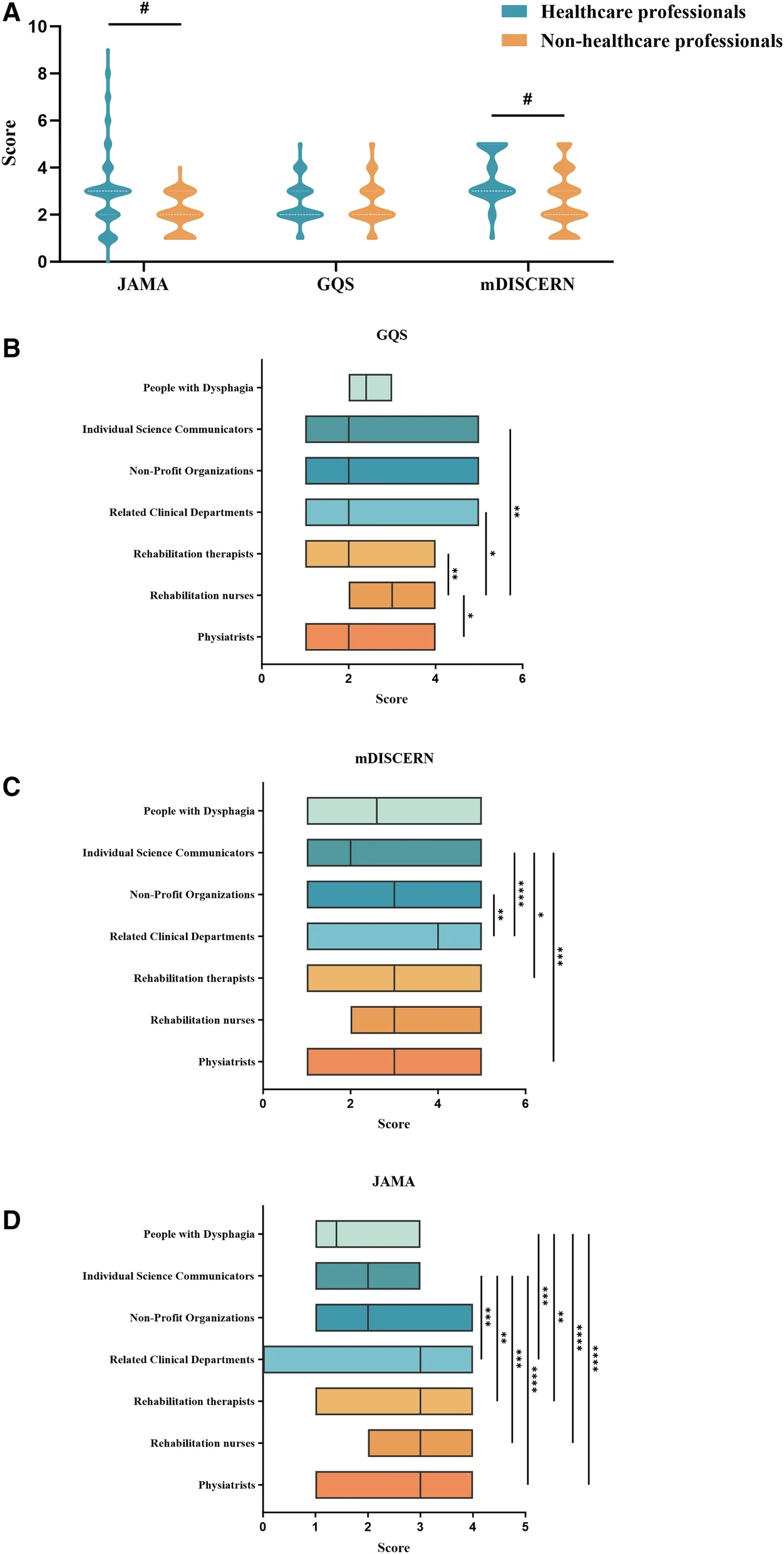
Compare GQS, JAMA, and mDISCERN scores of videos from different uploader sources **(A)** violin chart shows JAMA, GQS, and modified DISCREM scores for videos sourced from healthcare professionals and non-healthcare professionals. **#***P* *<* *.*05*.* **(B–D)** Box chart shows the video GQS, mDISCERN, and JAMA scores of 7 types of uploaders. *adjusted P *<* *.*05; **adjusted P *<* *.*01; ***adjusted P < .001; ****adjusted P < .0001.

Rehabilitation nurses scored the highest on the GQS and JAMA, whereas healthcare professionals in related clinical departments scored the highest on the mDISCERN (Table 6). For GQS scores, rehabilitation nurses scored significantly higher than physiatrists [adjusted *P* = .0324, *r* = .462, 95% CI (0.2, 0.66)], rehabilitation therapists [*adjusted P* = .001, *r* = .551, 95% CI (0.33, 0.71)], healthcare professionals in related clinical departments [*adjusted P* = .0259, *r* = .312, 95% CI (0.13, 0.47)], and individual science communicators [*adjusted P* = .004, *r* = .428, 95% CI (0.22, 0.6)] (Figure 6B).

For mDISCERN scores, individual science communicators scored the lowest and significantly lower than physiatrists [*adjusted P* < .001, *r* = .472, 95% CI (0.3, 0.62)], rehabilitation therapists [*adjusted P* = .0165, *r* = .336, 95% CI (0.15, 0.5)], and healthcare professionals in related clinical departments [*adjusted P* < .0001, *r* = .56, 95% CI (0.44, 0.66)] (Figure 6C).

For JAMA scores, individual science communicators and people with dysphagia scored significantly lower than rehabilitation nurses [*adjusted P* = .0001, *r* = .52, 95% CI [0.33, 0.67]; *adjusted P* < .0001, *r* = .937, 95% CI [0.86, 0.97]], physiatrists [*adjusted P* < .0001, *r* = .526, 95% CI [0.36, 0.66]; *adjusted P* < .0001, *r* = .73, 95% CI [0.55, 0.85]], rehabilitation therapists [*adjusted P* = .0029, *r* = .381, 95% CI [0.2, 0.54]; *adjusted P* = .0019, *r* = .559, 95% CI [0.33, 0.73]], and healthcare professionals in related clinical departments [*adjusted P* = .0001, *r* = .37, 95% CI [0.22, 0.5]; *adjusted P* = .0009, *r* = .405, 95% CI [0.23, 0.56]] (Figure 6D).

Analysis across different platforms revealed significant overall differences in GQS, JAMA, and mDISCERN scores (*H* = 12.74, df = 3, *P* = .0059, *ε*^2^ = .045; *H* = 34.26, df = 3, *P* < .0001, *ε*^2^ = .203; *H* = 139.3, df = 3, *P* < .0001, *ε*^2^ = .503). As shown in Figure 7 and Table 7, differences among platforms for GQS scores were generally small, with only Bilibili and Kwai showing a significant difference [*adjusted P* = .0034, *r* = .376, 95% CI (0.18, 0.55)]. For JAMA scores, Rednote, Douyin, and Bilibili were all significantly higher than Kwai [*adjusted P* < .0001, *r* = .387, 95% CI [0.23, 0.52]; *adjusted P* < .0001, *r* = .551, 95% CI [0.35, 0.64]; *adjusted P* = .0498, *r* = .288, 95% CI [0.08, 0.47]], and Douyin was significantly higher than Bilibili [*adjusted P* = .0014, *r* = .303, 95% CI (0.15, 0.44)]. For mDISCERN scores, Douyin had the highest scores, significantly higher than Rednote, Bilibili, and Kwai [*adjusted P* < .0001, *r* = .771, 95% CI [0.71, 0.82]; *adjusted P* < .0001, *r* = .757, 95% CI [0.68, 0.82]; *adjusted P* < .0001, *r* = .595, 95% CI [0.46, 0.71]], whereas no significant differences were observed among Rednote, Bilibili, and Kwai.

**Figure 7:**
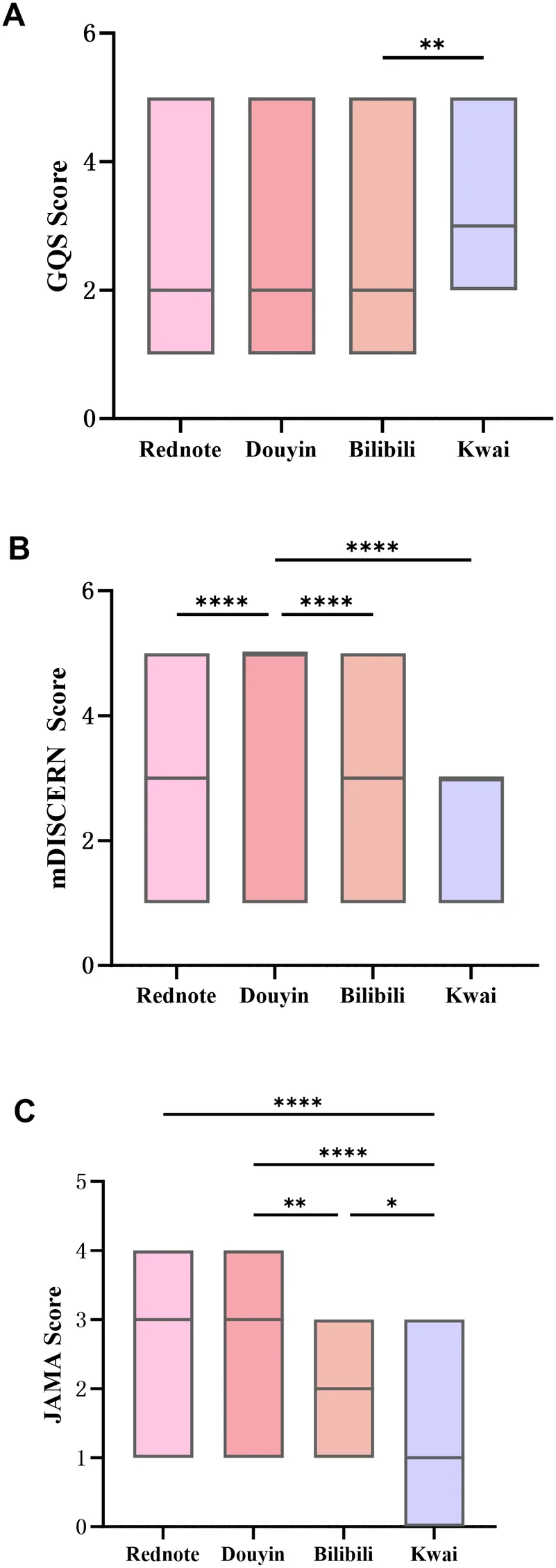
Compare GQS, JAMA, and modified DISCREM scores of videos from different platform sources. **(A–C)** Box charts display the GQS, modified DISCREM, and JAMA scores of the four short video platforms. *adjusted P *<* *.*05; **adjusted P *<* *.*01; ***adjusted P < .001; ****adjusted P < .0001.

**Table 7:** The GQS, JAMA, and mDISCERN scores of dysphagia feeding guidance videos by video platforms.

Video platform	Scores		
	GQS scores median (IQR)	JAMA scores median (IQR)	mDISCERN scores median (IQR)
Rednote	2 (2–3)	3 (2–3)	3 (2–3)
Douyin	2 (2–3)	3 (3–3)	5 (4–5)
Bilibili	2 (2–3)	2 (2–3)	3 (2–3)
Kwai	3 (2–4)	1 (1–2)	3(3–3)

Further analysis showed that for videos from Rednote and Douyin, JAMA scores were significantly lower among non-healthcare professionals than among healthcare professionals (*P* = .001; *P* < .001), whereas videos from Bilibili and Kwai showed no significant differences between healthcare professionals and non-healthcare professionals. For mDISCERN scores, healthcare professionals scored significantly higher than non-healthcare professionals for videos from Rednote, Douyin, and Bilibili.

(*P* < .001; *P* < .001; *P* = .01), but no significant difference was observed for videos from Kwai. For GQS scores, we found no significant differences between healthcare professionals and non-healthcare professionals across all platforms (Figure 8).

**Figure 8:**
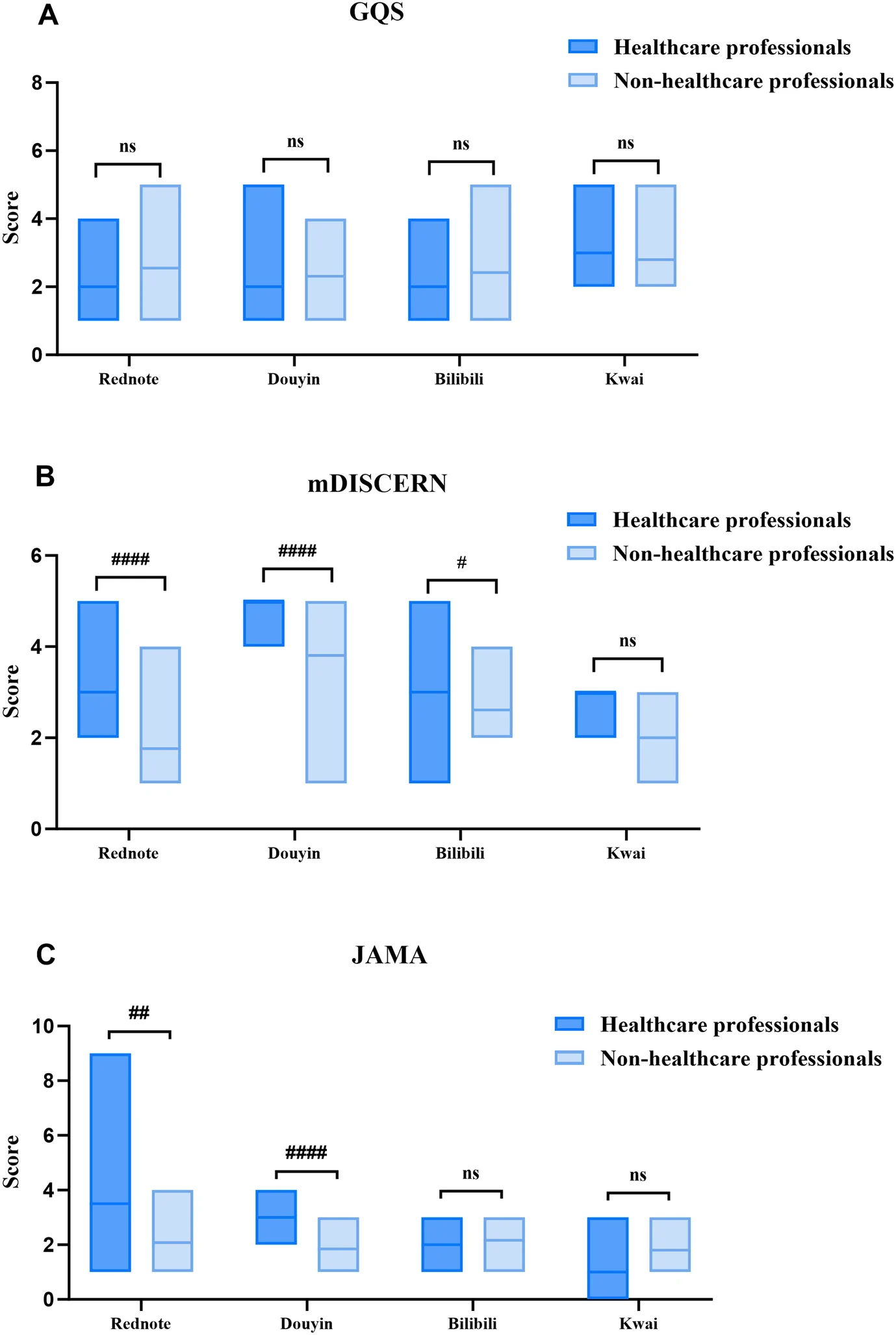
Compare the differences in (**A**) GQS, (**B**) modified DISCERN, and (**C**) JAMA scores of videos from healthcare professionals and non-healthcare professionals on four major platforms. **#***P* < .05; **##***P* < .01; **####***P* < .0001; ns: nonsignificant.

## 4 Discussion

### 4.1 Accessibility advantages and information quality risks of short videos for health communication

This study represents the first systematic evaluation of the overall quality, source transparency, information reliability, and domain coverage of feeding guidance videos for people with dysphagia across China's four major mainstream short-video platforms. Research findings indicate that over the past five years, short videos, with their intuitive, highly interactive, and easily disseminated characteristics, have contributed to an increase in the number of videos related to feeding guidance for people with dysphagia. This provides patients and caregivers with convenient access to self-management knowledge. Firstly, short videos visualize feeding guidance procedures (e.g.,) selecting appropriate thickeners to modify food consistency), making complex medical processes intuitively understandable. Second, videos uploaded by people with dysphagia themselves, representing real-life cases, enhance user interaction, lower barriers to knowledge acquisition, and promote the dissemination of health information.

However, the findings also reveal shortcomings in the dissemination of dysphagia health information via short videos. When searching using predefined keywords, over 58% of videos were irrelevant to the core topic. This finding may stem from inherent conflicts between algorithmic recommendation mechanisms and medical expertise (24) (e.g.,) confusing “swallowing” with entertainment content like food-eating challenges), low search accuracy due to keyword semantic generalization and content tagging abuse (25) (e.g.,) misclassifying numerous ordinary dining videos as relevant content), content creator ecosystems, and professional barriers (26) (e.g.,) a user base predominantly comprised of non-healthcare professionals), the professional limitations of platform content review mechanisms (e.g.,), reviewers lacking medical backgrounds), and the mismatch between user search behavior and platform responses (27) (e.g., General users often employ non-technical terminology for medical information searches, such as “choking while eating” instead of “dysphagia”).

Additionally, during the review of videos, it was found that most videos are subject to time constraints, resulting in overly simplified feeding guidance knowledge. For instance, key information such as indications, contraindications, and preparation methods for different food properties is often omitted, leading viewers to acquire only textbook-like concepts and struggle to apply them effectively in real-life scenarios. A study on the homogenization of information on YouTube during the early stages of the COVID-19 pandemic revealed that fragmented viewing patterns make it difficult for audiences to develop systematic cognition, as they acquire only isolated knowledge points while ignoring the overall medical logic, thereby undermining their ability to make correct health decisions (28). Meanwhile, algorithmic recommendation systems amplified the echo chamber effect, exposing users to persistently similar viewpoints and depriving them of opportunities to verify information from multiple perspectives. This increased the risk of misplaced trust in pseudoscience.

It is noteworthy that there is a mismatch between user engagement and the content's professional quality. Videos uploaded by rehabilitation therapists, though shorter in duration (median length of 67 s), received the highest median number of likes (65 times) and shares (34 times), indicating that if professional content can accurately meet user needs (such as practical skills), it can still achieve widespread dissemination. However, videos uploaded by non-healthcare professionals (such as patients) exhibit high engagement (median likes: 112) but low content reliability (median JAMA score: 1). Previous studies on health communication via short videos have also reported similar phenomena, wherein high interaction traffic coexists with low information quality (29, 30). This may be attributed to factors such as the algorithmic recommendation mechanisms of short-video platforms (31), the professional barriers to medical knowledge (32), the cognitive limitations of the target audience (33), the lag in regulatory frameworks (34), and the absence of standardized evaluation criteria (35). If such health education content dominates the information environment for people with dysphagia, the likelihood of patients encountering ineffective or even harmful feeding recommendations would increase significantly, thereby elevating the risk of aspiration pneumonia, medication errors, and unplanned readmissions.

### 4.2 Evaluation of the quality, source transparency, and information reliability of dysphagia feeding guidance information on short-video platforms: analysis using GQS, JAMA, and mDISCERN scores

In this study, we employed GQS, JAMA, and mDISCERN to assess the quality, source transparency, and information reliability of feeding guidance for people with dysphagia on short-video platforms. Given the high inter-rater agreement, all three assessment tools demonstrated good reliability and reproducibility in this sample.

#### 4.2.1 GQS score: overall information quality

Under the GQS-based evaluation framework (32), the overall quality, patient utility value, and content fluency of all video samples fell within the medium or lower ranges (median = 2, IQR = 2–3). Analysis by uploader identity revealed that GQS scores were largely comparable between healthcare uploaders and non-healthcare uploaders. Although the overall differences among the seven uploader categories were statistically significant, the effect sizes were only in the near-medium range, and most pairwise comparisons showed no statistically significant differences. This suggests that the overall educational quality of short videos does not vary significantly with the creator's professional identity; a professional background does not necessarily confer a perceptible advantage in information quality for end-users. Rehabilitation nurses demonstrated relatively superior performance on GQS scores, with robust medium effect sizes compared with physiatrists and rehabilitation therapists. Nursing work emphasizes health education, patient communication, and procedural demonstrations. This focus helps nurses break down complex clinical information into actionable steps. They also tend to present content in plain language, with visual aids and question-and-answer formats (36). A quality analysis of short health education videos in ophthalmology found that short-video accounts operated by nurses combine professional expertise with approachable content, a characteristic that leads to higher scores in standardized video quality assessment systems such as GQS (37).

Analysis by platform revealed that GQS scores differed significantly across the four platforms, but the effect size was only small to medium. After correction for multiple comparisons, only the pairwise comparison between Bilibili and Kwai showed a statistically significant between-group difference. Studies on other health topics have similarly shown that the magnitude of cross-platform differences reflected by GQS is limited and highly dependent on video content, information sources, and sample composition (38, 39). The present findings showed that video content in the field of dysphagia feeding management is highly concentrated on topics such as food texture modification, thereby narrowing differences in overall health information quality across platforms. Meanwhile, GQS scores do not directly reflect the authority of information sources or the rigor of evidence, thereby limiting GQS's sensitivity to platform ecosystem differences. Furthermore, algorithm-driven recommendation ranking may systematically affect the exposure of specific types of health information, leading to heterogeneity in the content pools encountered by users across platforms; such heterogeneity may not be fully captured by cross-sectional between-group comparisons.

#### 4.2.2 JAMA score: transparency of information sources

JAMA scores are used to evaluate the transparency of health information sources across four dimensions: author identity and qualifications, citation of sources, publication timeliness, and disclosure of conflicts of interest. The scores indicate whether a video includes the key elements that enable users to independently trace and verify the information (40). The present study showed that healthcare uploaders had significantly higher JAMA scores than non-healthcare uploaders (*P* < .001), and the overall differences among the seven uploader categories reached a medium effect size (*ε*^2^ = 0.203), suggesting that professional background exerts a substantial influence on source disclosure behavior. Studies of health information on YouTube have shown that videos uploaded by professionals tend to be higher quality and more reliable (41, 42). In addition, Sharon et al. (43) noted that although a large amount of misleading information exists on social media, users still place a high degree of trust in information from professionals. This implies that professional background affects not only the quality of information but also users' trust in it. Furthermore, rehabilitation nurses had the highest JAMA scores, whereas individual science communicators and people with dysphagia had the lowest scores, significantly lower than those of the four healthcare professional groups. This distribution may reflect the emphasis of nursing work on health education and patient guidance, which makes nurses more accustomed to clearly stating their identity, institutional affiliation, and information sources in their content, whereas patients and non-professional science communicators generally lack a systematic awareness of the necessity of source disclosure.

At the platform level, the JAMA scores of Rednote, Douyin, and Bilibili were significantly higher than those of Kwai, with effect sizes predominantly medium; among them, Douyin scored significantly higher than Bilibili. The cross-platform differences in information transparency may be attributable to differences in platform review and certification mechanisms. Regarding content review, Kwai imposes relatively weak mandatory requirements on source attribution and qualification verification for health information publishers, allowing content lacking proper authorship, standardized citations, and information disclosure to persist more readily. In contrast, Douyin has established creator certification and institutional account operation mechanisms that enable upfront disclosure of author identity, thereby improving the overall information transparency of platform content (44).

#### 4.2.3 mDISCERN score: information reliability

Based on the information reliability dimension of mDISCERN, the between-group differences were both statistically significant and robust. Healthcare professionals scored significantly higher on mDISCERN than non-healthcare professionals, suggesting that creators' professional identity, institutional affiliation, and authority of information sources were consistently associated with mDISCERN scores (45). Individual science communicators had the lowest scores, significantly lower than those of physiatrists, rehabilitation therapists, and healthcare professionals in related clinical departments, reflecting systematic deficiencies in core reliability dimensions, including content balance, source citation practices, and disclosure of uncertainty in non-professional science communication. Notably, the highest mDISCERN scores in this study were found among healthcare professionals in related clinical departments rather than rehabilitation nurses. This distribution pattern differs from the advantageous group distributions observed in the GQS and JAMA dimensions, indicating that different professional groups place different emphases across dimensions of health information quality.

The platform-level differences in mDISCERN scores were the most prominent among the three evaluation dimensions (*ε*^2^ = 0.503): Douyin's information reliability scores were significantly higher than those of the other three platforms, whereas no significant differences were observed among Rednote, Bilibili, and Kwai. These results suggest that, in the field of dysphagia feeding management, the influence of platform attributes on the reliability of health information is greater than their influence on overall information quality and source transparency. Douyin's advantage in information reliability may be associated with its creator composition, content review mechanisms, and the recommendation ecosystem for professional health content (23).

### 4.3 Domain coverage analysis of feeding guidance information for dysphagia on short-video platforms

#### 4.3.1 High coverage areas: the advantages of popularizing fundamental operational techniques

This study shows that food adjustment (77% coverage) and swallowing posture adjustment (52% coverage) are the two areas receiving the most attention. The rehabilitation nurse group among healthcare professionals has the highest score rate (food adjustment score rate of 100%; swallowing posture adjustment score rate of 80%). Food adjustment, a core component of dysphagia management, uses standardized food-texture classification systems to provide patients with safer, more nutritionally adequate dietary plans. This is beneficial not only for promoting the swallowing process and reducing the risk of aspiration and malnutrition, but also for improving the quality of life and rehabilitation outcomes (46, 47). Swallowing posture adjustment techniques directly optimize the biomechanical mechanisms of swallowing by altering head and neck positioning, and are commonly employed compensatory strategies in dysphagia feeding management (48). The “Guidelines for Rehabilitation Management of Swallowing Disorders in China (2023 Edition)” point out (14) that posture compensation during swallowing can change and move the direction of the swallowing channel, the size of the cavity, and the position of certain swallowing organ components, which can increase the sensory input stimulation of people with dysphagia, avoid aspiration and residue, and improve the occurrence of symptoms such as coughing. The basic operational techniques for dysphagia, such as food adjustment and swallowing posture adjustment, have a high coverage rate, reflecting the inherent advantages of short-video platforms in demonstrating specific operational skills. Food modifications (such as demonstrations of thickener use) and swallowing posture adjustments (such as head-position angle demonstrations) have high visual appeal and ease of replication, making them ideal content for short-form video platforms.

#### 4.3.2 Moderate coverage areas: the challenge of balancing professionalism and accessibility

Adjustments to eating methods (38% coverage), adjustment of feeding utensils (31% coverage), and feeding movement techniques (34% coverage) constitute the moderately covered group, with creators from different professional backgrounds exhibiting distinct group characteristics. Among healthcare professionals, rehabilitation nurses scored highest in eating utensil modifications and feeding techniques (80% and 73%, respectively). This is closely related to rehabilitation nurses' frequent involvement in patients' daily care during clinical practice. They are more familiar with using modified utensils, such as spill-proof bowls and spoons with thickened handles, to assist people with dysphagia in eating independently. Such practical experience gives them a unique advantage in tool adaptation. Among non-healthcare professionals, people with aphasia scored highest (80%) in the area of dietary adjustments, highlighting the unique value of experiential knowledge. Based on their long-term experience coping with dysphagia, patients are usually able to provide practical advice that is better aligned with their daily lives, such as the specific operation of intermittent oral-to-esophageal tube feeding at each meal, and the adjustment of eating rhythm under different physical states. These insights derived from the “user perspective” can precisely address details that healthcare professionals might overlook.

#### 4.3.3 Critical deficiencies: significant shortcomings in systemic management

Notably, five key areas were commonly overlooked: adjustments to the feeding environment, feeding risk control, oral care management, nutritional management, and medication management. The deficiency rates for these areas were 75%, 74%, 83%, 79%, and 96%, respectively. Feeding environment adjustment includes important measures such as reducing noise, enhancing lighting, and providing language prompts, which directly affect patients' attention allocation and swallowing coordination (49). Risk management during feeding primarily focuses on life-saving knowledge, such as identifying aspiration and performing choking rescue techniques (e.g., the Heimlich maneuver) (50). This systemic absence of safety management may cause patients and caregivers to focus solely on “how to eat” while neglecting “how to eat safely,” significantly increasing risks in home care. Oral care involves cleaning the oral mucosa, maintaining teeth and gums, and plays a crucial role in preventing infections (51). Nutritional management is directly related to the maintenance and improvement of patients' nutritional status and affects their long-term prognosis. The lack of such basic care knowledge may lead to frequent hospitalizations due to complications like oral infections and malnutrition, severely impacting patients' quality of life. The field of medication management is almost ignored by all video creators. However, the incidence of medication administration errors is significantly higher among people with dysphagia than among those without dysphagia (52). Medication management involves specialized procedures such as crushing, dissolving, or mixing medications with food, which directly determine the safety and efficacy of pharmacotherapy (53). From a pharmacokinetic standpoint, crushing extended-release or enteric-coated formulations may trigger dose dumping or drug inactivation, leading to acute toxicity or therapeutic failure (54). From a clinical outcomes standpoint, these pharmacokinetic disturbances can further precipitate severe hypotension, cardiovascular collapse, severe hypoglycemia, respiratory depression, seizures, aspiration pneumonia, and even death. At the healthcare system level, such adverse outcomes increase the rates of emergency department visits, hospitalizations, and ICU admissions, as well as overall healthcare expenditures (55). For patients and their families, these outcomes cause additional suffering, may lead to disability, and substantially diminish quality of life. Notably, however, such adverse outcomes are largely preventable. Given that family caregivers play a central role in medication management for people with dysphagia, providing them with standardized educational interventions is of considerable practical importance. Previous studies have demonstrated that targeted educational interventions can reduce the rate of inappropriate medication crushing from 3.1% to 0.5% (56).

### 4.4 Implications for clinical practice and health communication

As short-video platforms have increasingly become a key channel through which people with dysphagia and their caregivers obtain feeding guidance information, they present considerable value for health education but also pose risks to information quality. The present study found significant differences across platforms in both information quality and source transparency. It also revealed insufficient coverage of key content domains, such as medication management and nutritional management, as well as considerable variation in content quality across uploader groups. Accordingly, coordinated efforts are required across clinical practice, platform governance, and evaluation systems to maximize the positive contributions of short videos to dysphagia feeding management.

At the clinical practice level, clinicians should proactively integrate short-video resources into patient education. Specifically, professionals such as physiatrists and speech-language pathologists may recommend certified videos that have undergone quality assessment based on individual patient conditions, thereby effectively extending health education beyond traditional medical settings. Previous studies have demonstrated that video-based education can enhance the effectiveness of health information dissemination through a more intuitive format (57–59). The present findings also indicate that creators from different professional backgrounds vary in their content coverage, highlighting the need to establish a multidisciplinary content-creation ecosystem that leverages the strengths of diverse professionals. On this basis, healthcare professionals should adapt to the communication dynamics of new media while ensuring scientific rigor, drawing on narrative techniques employed by non-professional creators, and prioritizing science communication in underserved domains such as medication management and nutritional support. Furthermore, healthcare professionals should provide patients and caregivers with lists of quality-assessed videos or certified creator accounts, with particular emphasis on core points such as feeding risk control, to reduce exposure to low-quality health information. Medication management, in particular, should constitute a central component of targeted health education. Although patient-uploaded content may offer certain experiential value, such as in adjusting feeding methods, it often lacks source transparency and information reliability. It is therefore recommended that healthcare professionals participate in content review or joint creation to preserve experiential value while safeguarding information quality.

At the platform governance level, platform operators should improve content review mechanisms for health-related content and establish a creator qualification certification system. This study found that platforms with lower source transparency generally exhibit inadequate disclosure of author identities, citation information, and conflicts of interest. It is therefore recommended that qualification verification and disclosure requirements be implemented prior to content publication, and that health-related science communication accounts be subject to tiered management. For professional videos that pass the review, explicit labels such as “medical certification” should be added. Recommendation algorithms should also incorporate quality and safety weighting to prioritize content that covers risk management and clearly cites sources, thereby preventing high-engagement but low-reliability videos from being over-promoted based on their completion rates and like counts. In parallel, differentiated content delivery may help mitigate filter bubble effects. With regard to search functions, platforms should establish semantic mappings between everyday language and standardized medical terminology. For example, associating lay search terms such as “choking while eating” with standardized terms such as “dysphagia” and “aspiration” can reduce users' exposure to irrelevant content.

At the evaluation system level, a multidimensional assessment mechanism encompassing information quality, transparency of information sources, information reliability, and domain coverage should be established to support tiered labeling of health science communication videos. Given that no single assessment tool can fully capture the health education value of videos, the combined use of multiple instruments such as GQS, JAMA, and mDISCERN can provide evidence-based support for clinical recommendations, platform governance, and content optimization by creators. Through such coordinated multi-level interventions, the positive contribution of short videos to dysphagia feeding management can be maximized while ensuring the scientific integrity of the information.

## 5 Limitations and prospects

First, this study employed a sampling strategy based on platform recommendation ranking, which closely approximates the highly engaging content that users are likely to encounter in routine use. However, this approach may omit high-quality early videos that received low exposure. In addition, the dynamic and personalized nature of recommendation algorithms makes search results difficult to reproduce, and videos removed for policy violations or proactively deleted by creators cannot be included in the sample. These factors inevitably limit the representativeness of the sample. Future studies may mitigate such algorithmic sampling bias by adopting preregistered search protocols, standardized search accounts, and longitudinal data collection within fixed time windows, or by collaborating with platforms to access de-identified open data.

Second, the sample sizes were unevenly distributed across platforms, and the subgroup sample sizes defined by uploader identity were relatively small, which may have reduced the statistical power of the subgroup analyses. Future studies should increase sample sizes across all uploader categories and conduct more rigorous validation of uploader classification results.

Third, the domain coverage score used in this study was developed by our research team and has not been externally validated for reliability and validity. For engagement metrics, although video age (i.e., days since publication) was recorded. This variable was not included in the statistical adjustments, nor were raw counts such as likes, comments, favorites, and shares converted into time-standardized indicators [(e.g.,) daily interaction counts]. Cross-platform comparisons of these raw metrics are further complicated by differences in user base size, traffic distribution mechanisms, and platform functionality. Because earlier-published videos may accumulate higher absolute engagement counts simply due to longer dissemination times, these confounding factors may compromise the accurate assessment of the relationship between user engagement and content quality. To address these issues, future studies could develop assessment tools tailored to the characteristics of short-video media and the specific requirements of the dysphagia domain, including dissemination metrics such as video age and exposure as covariates, and adopt standardized engagement metrics [(e.g.,) daily like counts, and interaction rates based on view counts] to enhance cross-platform comparability, and conduct external validation of the scoring system.

Finally, and most importantly, this study was confined to Chinese short-video platforms, and the cross-sectional design could only reflect the health education content landscape of dysphagia feeding guidance as of September 2025, without capturing ongoing content updates or platform policy changes, nor obtaining actual patient viewing behaviors or usability evaluation data. Given that the GQS, JAMA, and mDISCERN assess only content quality, the potential effects of such content on audience behavioral outcomes cannot be captured; therefore, causal inferences regarding the impact of short-video health content on clinical outcomes such as aspiration pneumonia, medication administration errors, and readmissions cannot be drawn. Future research could develop instruments specifically tailored to analyzing video quality in the field of dysphagia feeding guidance, conduct cross-language and cross-platform comparative studies, and incorporate longitudinal cohort designs or viewing log analyses, thereby directly examining the pathways through which content quality influences patients' knowledge, cognitive attitudes, self-management behaviors, and clinical prognosis. Furthermore, promoting transparency in platform algorithms and establishing a multi-stakeholder collaborative governance system are core directions for optimizing the effectiveness of health communication through evidence-based mechanisms.

## 6 Conclusion

The present study systematically evaluated 278 short videos addressing feeding guidance for dysphagia across four major Chinese short-video platforms: Rednote, Douyin, Bilibili, and Kwai. The results indicated that the overall quality of videos in this field was moderate or below, with marked structural imbalances in content coverage. The influence of the uploader's professional background on information quality varied by assessment dimension: medical uploaders significantly outperformed non-medical uploaders in source transparency and information reliability, whereas uploaders from different professional backgrounds exhibited distinct strengths and weaknesses across dimensions. Platform-level quality differences were likewise dimension-dependent. Differences in information reliability were the most pronounced, with Douyin scoring significantly higher than the other platforms, whereas differences in overall information quality were relatively limited. In addition, a subset of high-engagement videos in this study received low information reliability scores, suggesting that engagement metrics should not be used as a standalone indicator of information reliability.

In summary, short videos provide people with dysphagia a convenient and intuitive channel for accessing feeding guidance. However, the current content ecosystem in this field remains insufficient to support informed decision-making and proper self-management among this population. Fostering a high-quality environment for dysphagia-related health communication requires coordinated efforts across professional healthcare involvement, platform governance, quality assessment mechanisms, and user health literacy education.

## Data Availability

The original contributions presented in the study are included in the article/Supplementary Material, further inquiries can be directed to the corresponding author/s.
