# Supplementary material for: Quality, source transparency, information reliability, and domain coverage of short video-based information on feeding guidance for people with dysphagia: a cross-sectional study on 4 major Chinese social media platforms
Source: Front Digit Health. 2026 Oct 14;8:1919212. doi: 10.3389/fdgth.2026.1919212 (PMC13713780; doi:10.3389/fdgth.2026.1919212)
Supplement: Supplementary file 1 [file Table1.docx]

Supplementary table 2. The Journal of the American Medical Association (JAMA) benchmark criteria.

| Score* | Score component | |
| --- | --- | --- |
| 1 score | Authorship | Provided authorship information |
| 1 score | Attribution | Listed copyright information and references/sources |
| 1 score | Currency | Included the initial date and subsequent updates |
| 1 score | Disclosure | Disclosed any potential conflicts of interest, funding, sponsorship, advertising support or video ownership |

*The criteria of each aspect were scored separately, and 1 point was accumulated when the criteria were reached. A total reliability score ranging from 0 to 5 was obtained.
