# Supplementary material for: Quality, source transparency, information reliability, and domain coverage of short video-based information on feeding guidance for people with dysphagia: a cross-sectional study on 4 major Chinese social media platforms
Source: Front Digit Health. 2026 Oct 14;8:1919212. doi: 10.3389/fdgth.2026.1919212 (PMC13713780; doi:10.3389/fdgth.2026.1919212)
Supplement: Supplementary file 2 [file Table2.docx]

Supplementary table 3. Modified DISCERN benchmark criteria.

| Score* | Reliability Score |
| --- | --- |
| 1 score | The video was clear, concise and understandable |
| 1 score | The information sources were reliable |
| 1 score | The information presented was balanced and unbiased |
| 1 score | Additional sources of information were provided for patient reference |
| 1 score | Areas of uncertainty or controversy were appropriately addressed. |

*The criteria of each aspect were scored separately, and 1 point was accumulated when the criteria were reached. A total reliability score ranging from 0 to 5 was obtained.
