# Supplementary material for: Quality, source transparency, information reliability, and domain coverage of short video-based information on feeding guidance for people with dysphagia: a cross-sectional study on 4 major Chinese social media platforms
Source: Front Digit Health. 2026 Oct 14;8:1919212. doi: 10.3389/fdgth.2026.1919212 (PMC13713780; doi:10.3389/fdgth.2026.1919212)
Supplement: Supplementary file 3 [file Table3.docx]

Supplementary table 1. Global Quality Score (GQS) benchmark criteria.

| Score | Global Score Description |
| --- | --- |
| 1 score | poor quality, poor traffic, most information missing, not of any use to the patient |
| 2 score | Generally poor quality with poor flow, some information listed but many important topics lacked, of very limited use to patients |
| 3 score | medium quality with suboptimal flow, some of the main information was fully discussed but other information discussed insufficiently, somewhat helpful to patients. |
| 4 score | Good quality and generally goo flow, most of the relevant information is listed, but some topics not covered, useful to patients. |
| 5 score | Excellent quality and excellent flow, very useful for patients |
