# Supplementary material for: Quality, source transparency, information reliability, and domain coverage of short video-based information on feeding guidance for people with dysphagia: a cross-sectional study on 4 major Chinese social media platforms
Source: Front Digit Health. 2026 Oct 14;8:1919212. doi: 10.3389/fdgth.2026.1919212 (PMC13713780; doi:10.3389/fdgth.2026.1919212)
Supplement: Supplementary file 4 [file Table4.docx]

**Supplementary Table 4. Inter-rater reliability of the three instruments**

| Instrument  (scale) | n | Quadratic weighted κ (95% CI) | Unweighted κ (95% CI) | ICC(2,1) (95% CI) | Exact agreement | Adjudication (≥2-grade) |
| --- | --- | --- | --- | --- | --- | --- |
| GQS  (1–5) | 278 | 0.860  (0.806–0.907) | 0.830  (0.771–0.884) | 0.893  (0.844-0.937) | 88.8% | 6/278 (2.2%) |
| JAMA  (0–4) | 278 | 0.873  (0.825–0.914) | 0.843  (0.788–0.893) | 0.906  (0.861-0.942) | 89.9% | 3/278 (1.1%) |
| mDISCERN  (0–5) | 278 | 0.884  (0.845–0.919) | 0.811  (0.753–0.864) | 0.936  (0.908-0.959) | 86.0% | 5/278 (1.8%) |

| Instrument | Source | n | Weighted κ | Unweighted κ | ICC(2,1) |
| --- | --- | --- | --- | --- | --- |
| GQS | Rednote | 110 | 0.842 | 0.821 | 0.868 |
| GQS | Douyin | 84 | 0.869 | 0.837 | 0.911 |
| GQS | Bilibili | 63 | 0.854 | 0.813 | 0.899 |
| GQS | Kwai | 21 | 0.881 | 0.865 | 0.910 |
| JAMA | Rednote | 110 | 0.868 | 0.838 | 0.910 |
| JAMA | Douyin | 84 | 0.897 | 0.860 | 0.925 |
| JAMA | Bilibili | 63 | 0.768 | 0.739 | 0.805 |
| JAMA | Kwai | 21 | 0.949 | 0.927 | 0.971 |
| DISCERN | Rednote | 110 | 0.847 | 0.780 | 0.908 |
| DISCERN | TikTok | 84 | 0.830 | 0.801 | 0.878 |
| DISCERN | Bilibili | 63 | 0.683 | 0.620 | 0.764 |
| DISCERN | Kwai | 21 | 0.907 | 0.868 | 0.944 |
